# Indicaxanthin Induces Autophagy in Intestinal Epithelial Cancer Cells by Epigenetic Mechanisms Involving DNA Methylation

**DOI:** 10.3390/nu15153495

**Published:** 2023-08-07

**Authors:** Maria Antonietta Ragusa, Flores Naselli, Ilenia Cruciata, Sara Volpes, Chiara Schimmenti, Graziella Serio, Maurizio Mauro, Mariangela Librizzi, Claudio Luparello, Roberto Chiarelli, Chiara La Rosa, Antonino Lauria, Carla Gentile, Fabio Caradonna

**Affiliations:** 1 Department of Biological, Chemical and Pharmaceutical Sciences and Technologies (STEBICEF), University of Palermo, 90128 Palermo, Italy; maria.ragusa@unipa.it (M.A.R.); flores.naselli@unipa.it (F.N.); ilenia.cruciata@unipa.it (I.C.); sara.volpes@unipa.it (S.V.); chiara_schimmenti@libero.it (C.S.); graziella.serio01@unipa.it (G.S.); librizzimariangela87@gmail.com (M.L.); claudio.luparello@unipa.it (C.L.); roberto.chiarelli@unipa.it (R.C.); antonino.lauria@unipa.it (A.L.); fabio.caradonna@unipa.it (F.C.); 2 Department of Obstetrics & Gynecology and Women’s Health, Michael F. Price Center, Albert Einstein College of Medicine, Bronx, NY 10461, USA; maurizio.mauro5@gmail.com; 3 NBFC—National Biodiversity Future Center, 90133 Palermo, Italy; 4 Department of Life Sciences and Systems Biology, Neuroscience Institute Cavalieri Ottolenghi, University of Torino, 10124 Turin, Italy; chiara.larosa.88@gmail.com

**Keywords:** acidic vesicular organelles, bioactive compounds, Caco-2, cell biology, DNA methylome, epigenetics, gene expression, nutrigenomics, *Opuntia ficus indica*, reduced representation bisulphite sequencing

## Abstract

Autophagy is an evolutionarily conserved process critical in maintaining cellular homeostasis. Recently, the anticancer potential of autophagy inducers, including phytochemicals, was suggested. Indicaxanthin is a betalain pigment found in prickly pear fruit with antiproliferative and pro-apoptotic activities in colorectal cancer cells associated with epigenetic changes in selected methylation-silenced oncosuppressor genes. Here, we demonstrate that indicaxanthin induces the up-regulation of the autophagic markers LC3-II and Beclin1, and increases autophagolysosome production in Caco-2 cells. Methylomic studies showed that the indicaxanthin-induced pro-autophagic activity was associated with epigenetic changes. In addition to acting as a hypermethylating agent at the genomic level, indicaxanthin also induced significant differential methylation in 39 out of 47 autophagy-related genes, particularly those involved in the late stages of autophagy. Furthermore, in silico molecular modelling studies suggested a direct interaction of indicaxanthin with Bcl-2, which, in turn, influenced the function of Beclin1, a key autophagy regulator. External effectors, including food components, may modulate the epigenetic signature of cancer cells. This study demonstrates, for the first time, the pro-autophagic potential of indicaxanthin in human colorectal cancer cells associated with epigenetic changes and contributes to outlining its potential healthy effect in the pathophysiology of the gastrointestinal tract.

## 1. Introduction

Autophagy is implicated in maintaining the balance between the synthesis, degradation, and recycling of cellular components. This process involves the formation of an autophagosome, endowed with a double membrane enclosing cytoplasmic constituents, that subsequently fuses with a lysosome to generate a mature autophagolysosome, in which cellular components are degraded and subsequently released.

Both the impairment and excessive activation of autophagy are correlated with multiple pathophysiological states and ageing [1], and much scientific evidence seems to indicate that the activation of autophagy may be positive for health and longevity [2]. Among the strategies for autophagy activation, interest in nutritional behaviours is growing. Scientific data suggest that a ketogenic diet, caloric restriction, and intermittent fasting can modulate autophagy at the systemic level [3]. Additionally, several food ingredients have been found to be able to influence various pathways in the course of autophagy [4].

The role of autophagy in cancer is complex and debated. Scientific evidence shows that autophagy allows cell survival under conditions of hypoxia and nutrient deficiency. As these conditions are common in the microenvironment of solid tumours, autophagy can support the survival of cancer cells [5]. Indeed, by degrading and recycling cellular components, autophagy can provide the energetic substrates to cells necessary to survive under conditions of paucity of nutrients and external energy sources [6]. In contrast, other scientific data indicate that autophagy can promote tumour suppression, although the mechanism is not yet clear. By preventing the accumulation of damaged cellular components, autophagy may reduce reactive oxygen species production, thus avoiding DNA mutations and genomic instability. Autophagy can also inhibit cellular proliferation by promoting cellular senescence [7]. Finally, the self-propagation of autophagy can trigger cell death processes due to the excessive degradation of cellular constituents [8]. Indeed, autophagy has also been described as a “type II programmed cell death mechanism”, a process that can flow with the more classical apoptotic death (type I programmed cell death) or just replace it [9]. Collectively, it has been suggested that the ambiguous role of autophagy, which can promote or inhibit tumour formation, may depend on the type of cancer and the different stages in which it is acting [10].

Several studies in mammals have shown the importance of some autophagy genes in tumour suppression. In fact, it was demonstrated that the specific miRNA-mediated loss of autophagy, or the loss of genes responsible for autophagy [7,11], may contribute to tumorigenesis [12]. For example, the monoallelic loss of *BECN1*, coding for the autophagy regulator Beclin1, leads to increased spontaneous breast, ovarian, and prostate tumorigenesis in murine models [13]. Conversely, the activation of this gene inhibits tumour cell growth in vitro and tumour formation in vivo [14].

Experimental evidence shows that many phytochemicals possess chemotherapeutic properties and some of them have been characterized for their ability to induce autophagy [15,16]. These effects may be of interest in the context of the intestinal pathologies or even physiopathological states of the gastro-enteric tract. Recent studies have, in fact, shown that autophagy is involved in the modulation of intestinal inflammation, indicating that autophagy is able to alleviate inflammatory bowel diseases through the crosstalk of specific genes [17]. Moreover, the gut exerts immune regulation by a mechanism involving autophagy phenomena of intestinal epithelial cells [18].

Betalains are an emerging class of phytochemicals. Their distribution is restricted to nine of the twelve families of the Cariophillales order, and beetroot (*Beta vulgaris*) and fruits of *Opuntia* species, such as *Opuntia ficus indica*, are the main sources of these molecules. The antitumoral potential of betalains has been explored through in vitro and in vivo models [19]. The yellow pigment from *Opuntia ficus indica*, indicaxanthin (IND), is highly bioavailable in humans in its native form [20]. Scientific data have shown that IND possesses free radical scavenging and antioxidant activities [21] and demonstrates anti-inflammatory activities in intestinal epithelial cells in vitro and in murine models of inflammation [22]. Moreover, we showed that IND exhibited cytotoxic activity in several colorectal cancer cell lines, including Caco-2 [23,24].

The human epithelial cell line Caco-2 is a well-established and widely used model of the intestinal epithelium. Caco-2 cells derive from a human adenocarcinoma and are able to spontaneously differentiate into a mature enterocyte-like phenotype when cultured about two weeks after confluence [25]. Differentiated Caco-2 cells exhibit a normal-like phenotype and have been used as a valuable model for studying intestinal uptake and the transport of nutrients and drugs [26], and have found application in the study of several cellular processes related to the small intestine’s pathophysiology [27].

Previously, we showed that IND did not affect the viability of differentiated Caco-2 cell monolayers, but possessed antiproliferative activity in proliferating cells associated with the epigenetic modulation of some methylation-silenced oncosuppressor genes, including *p16^INK4a^* [23,24]. We also demonstrated that IND influenced the expression of DNA methyltransferases (DNMTs) in proliferating Caco-2 cells and was able to affect their activity by binding the catalytic sites [23].

In this work, we evaluated the effects of IND on the autophagic process in colon adenocarcinoma epithelial cells by in vitro and in silico approaches and using *omics* methods.

## 2. Materials and Methods

### 2.1. Cell Culture and Treatments

The Caco-2 colon adenocarcinoma cell line was cultured in high-glucose–DMEM medium plus 10% foetal calf serum (ThermoFisher, Waltham, MA, USA), 100 U/mL penicillin, 100 µg/mL streptomycin, and 2.5 mg/L amphotericin B (Invitrogen, Carlsbad, CA, USA) at 37 °C under a 5% CO_2_ atmosphere, as described by Mauro et al. (2013) [28], with some modifications reported by Librizzi et al. (2015) [29].

IND was isolated from *Opuntia ficus indica* fruit extracts, as previously reported [30]. Caco-2 cells were exposed to different concentrations of IND, i.e., 10 (IND10), 50 (IND50), and 100 µM (IND100), for 48 h. Ten micromoles of 5-aza-2 deoxycytidine (5-azaC) were used as a positive control. Considering the half-life of 5-azaC, an equal fresh quantity was added after the first 24 h of exposure. These experiments were performed in duplicate.

### 2.2. Western Blotting

Proteins were extracted and Western blotting was performed as described by Librizzi et al. (2015) [29] and Caradonna et al. (2018) [31]. Samples were reacted with the following primary antibodies: anti-LC3 (Sigma-Aldrich, Waltham, MA, USA, #L8918, 1:750); anti-Beclin1 (Santa Cruz Biotecnology, Dallas, TX, USA, #11427, 1:500); anti–α-tubulin (Sigma-Aldrich, #T5168, 1:500); and peroxidase-conjugated anti-mouse or anti-rabbit secondary antibodies (Promega Corporation, Madison, WI, USA, #W4021 and #W4011, 1:10000). The chemiluminescent signals were revealed by Chemidoc XRS (Bio-Rad, Hercules, CA, USA) using the SuperSignal™ West Pico PLUS Chemiluminescent Substrate (Thermo, Waltham, MA, USA, #34580). The protein expression data were normalized using α-tubulin band intensity as the loading control. The quantitative data were reported as bar plots derived from the densitometric scans of the bands obtained after at least three Western analyses using the ImageJ software (v. 1.53t).

### 2.3. Acidic Vesicular Organelles Detection by Flow Cytometry

The detection and quantitation of acidic vesicular organelles and autophagy markers were evaluated by flow cytometry, as reported by Luparello et al. (2019) [32]. Briefly, Caco-2 cells, both untreated and those treated with 10, 50, or 100 µM IND, were collected and stained with acridine orange (final concentration 100 μg/mL), and then analysed using a FACSCanto flow cytometer. The increase in red fluorescence intensity was indicative of the increment in the number of autophagolysosomes. These experiments were performed in triplicate.

### 2.4. Methylomic Studies

#### 2.4.1. Reduced Representation Bisulphite Sequencing (RRBS) and Differential Methylation Analysis

The RRBS approach is now becoming increasingly common because it allows genome-scale DNA methylation analysis in a highly accurate and low-cost manner. Indeed, the fragments obtained from RRBS include most promoters, as well as repeated sequences (which often contain methylated cytosines), which are difficult to profile using conventional approaches. The samples were prepared according to the Diagenode Premium RRBS Kit protocol and sequenced on an Illumina HiSeq platform using 150 bp paired-end reads. The raw FASTQ reads were initially quality-tested using FastQC (https://www.bioinformatics.babraham.ac.uk/projects/fastqc/ accessed on 30 January 2023). Subsequently, they were aligned against the reference human genome (GRCh38/hg38) using the bisulphite-specific short-read aligner BSMAP v 2.90 [33]. The restriction enzyme digestion site parameter was set to ‘C-CGG’ for *Msp*I digestion. Following alignment, the BAM files were sorted and indexed using SAMtools [34]. The methylation ratio on individual samples was calculated using the BSMAP v 2.90 methratio script. The company that carried out the RRBS technique (Galseq s.r.l.) delivered a series of typical methylation call files containing the data and software for basic analysis called “RRBS viewer”, which allowed the visualization of the data. The data quality assessment was performed using the R (version 4.0.3) package *methylKit* and bimodal CpG methylation % profiles were obtained. The general coverage statistics were checked and the samples were filtered based on coverage (minimum coverage <10 and >99.9th percentile of coverage in each sample). The mean coverage obtained on these CpG sites ranged from 22 to 26 between five methylomes.

#### 2.4.2. Differentially Methylated Cytosine (DMC) Method Analysis (Individual CpG Method)

At the gene level, those mainly involved in each phase of autophagy were selected and differential analysis was performed on both individual CpGs and 200 bp tiles. For each gene, the RRBS viewer was used (reference datasets Gencode Release 29, assembly GRCh38/hg38) to examine the differential methylation profile following cell treatments. As the output, RRBS viewer provides log2 differential methylation data of each CpG contained in the gene region. The region that extended from −1000 to +1000 bp with respect to the transcription start site (TSS) was considered as the “promoter”. To obtain the latest annotation version, the data were downloaded using the Table Browser tool from the UCSC (University of California Santa Cruz) Genome Browser. For some autophagy genes that possess two TSSs, analyses were also carried out on these additional promoters (for simplicity, these genes will be identified with the name of the gene followed by a dot and number 2). The complete list of selected genes and chromosomal ranges corresponding to their promoters and gene bodies are indicated in Table S1. The average was calculated for each promoter region and the variation was considered significant if |diff.meth| was ≥0.4 (at least 25%).

To cluster the samples based on the similarity in the methylation variation, the heatmap function of R was used and similarity groups were defined.

#### 2.4.3. Tile-Based Method Analysis

To observe the changes in the degree of methylation relative to the regions and, therefore, to better highlight the differences in methylation between the treated and untreated samples, the differentially methylated regions (DMRs) were also determined by *methylKit* pairwise comparison using a tile-based method. Tile-based analysis affords higher statistical power due to the aggregation of the signals from multiple CpGs within a defined genomic region. *MethylKit* differential analysis was performed at the level of 200 bp tiles using default parameters: *q*-value < 0.01 and minimum coverage in the tile equal to 10. Tiles with |diff.meth| greater than 25% were considered, as lower values were not indicative of substantial differences.

Using the R package *genomation*, the regions corresponding to the selected tiles were then annotated to assess whether they corresponded to specific regulation elements (cCRE, ORegAnno, and CpGI tracks). The analysis of the tiles was carried out for the regions corresponding to both promoters and gene bodies.

### 2.5. Local DNA Methylation Assessment by Methylation-Sensitive Restriction Endonuclease–PCR (MSRE-PCR)

The isolation of genomic DNA from Caco-2 cells was carried out as described by Longo et al. [35] and its quantitation was obtained with the NanoDrop microvolume sample retention system ND-1000 (Thermo Scientific NanoDrop Products).

To evaluate the differential methylation status of the *BECN1* gene promoter after cell treatment with IND, the MSRE-PCR technique was used [36]. This approach is based on the inability of some restriction enzymes to cut DNA sequences that contain one or more methylated cytosines and, consequently, to permit or not PCR amplification. Firstly, a 669 bp CpG island was identified in the promoter of the *BECN1* gene (Human Assembly hg38 coordinates: chr17:42,823,622-42,824,291). In this region, those primer couples that allowed the amplification of four fragments, each containing unique CpG sites for *Hpa*II and *Hha*I methylation-sensitive restriction enzymes, were chosen (Table S2). A semi-quantitative PCR protocol was applied, carrying out a low number of cycles and, therefore, taking data from the exponential phase of the PCR amplification. After the PCR reactions, every sample was analysed in a 6% polyacrylamide gel, and fragments of all the expected sizes were observed. These experiments were performed in duplicate.

### 2.6. In Silico Analysis of Molecules Interactions

To gain more insight into the capability of IND to form a complex with Bcl-2 at the Beclin1 binding site, molecular modelling studies were performed, starting from the identification of the 3D structures of the Bcl-2-Beclin1 complex available in the Protein Data Bank database [37].

#### 2.6.1. Protein Preparation

The crystal structure of Bcl-2 complexed with Beclin1 (PDB id 5VAU) was downloaded from the Protein Data Bank [37]. The Protein Preparation Wizard in Schrödinger 2023-1 software was subsequently employed for the further preparation of the protein structure using the default settings [38]. Bond orders were assigned, and hydrogen atoms, as well as protonation of the heteroatom states, were added using the Epik-tool (with the pH set at biologically relevant values, i.e., at 7.0 ± 0.4). The H-bond network was then optimized. The structure was subjected to a restrained energy minimization step (RMSD of the atom displacement for terminating the minimization was 0.3 Å) using the Optimized Potentials for Liquid Simulations (OPLS) 2005 force field [39].

#### 2.6.2. Ligand Preparation

The default setting of the LigPrep tool implemented in Schrödinger’s software (version 2017-1) was used to prepare IND for molecular docking [40]. All possible tautomers and combinations of stereoisomers were generated for pH 7.0  ±  0.4 using the Epik ionization method [41]. Energy minimization was subsequently performed using the integrated OPLS 2005 force field [39].

#### 2.6.3. Induced Fit Docking (IFD) and Molecular Dynamic Simulation

IFD simulation was performed using the IFD application available [42,43] in the Schrödinger software (Schrodinger 2023-1) suite, which has been demonstrated to be an accurate and robust method to account for both ligand and receptor flexibility [44].

The IFD protocol was performed as follows [45,46]: IND was docked into the rigid receptor model with scaled-down van der Waals (vdW) radii. The Glide Standard Precision (XP) mode was used for the docking and 20 ligand poses were retained for protein structural refinements. The docking boxes were defined to include all amino acid residues within the dimensions of 40 Å × 40 Å × 40 Å from the centre of the original ligand. The induced-fit protein–ligand complexes were generated using Prime software (Schrödinger Release 2023-3) [47,48]. The 20 structures from the previous step were submitted to side-chain and backbone refinements. All residues with at least one atom located within 5.0 Å of each corresponding ligand pose were included in the refinement by Prime. All the poses generated were then hierarchically classified, refined, and further minimized into the active site grid before finally being scored using the proprietary GlideScore function defined as follows: GScore = 0.065 × vdW + 030 × Coul + Lipo + Hbond + Metal + BuryP + RotB + Site; where vdW is the van der Waals energy term; Coul is the Coulomb energy; Lipo is a lipophilic contact term that rewards favourable hydrophobic interactions; Hbond is an H-bonding term; Metal is a metal-binding term (where applicable); BuryP is a penalty term applied to buried polar groups; RotB is a penalty for freezing rotatable bonds; and Site is a term used to describe favourable polar interactions in the active site.

Finally, the IFD score (IFD score = 1.0 Glide_Gscore + 0.05 Prime_Energy), which accounts for both the protein–ligand interaction energy and total energy of the system, was calculated and used to rank-select the best IFD pose. To assess the complex stability and to dissect the amino acids involved in the interaction, the IFD best-scored output was submitted to 20 ns of molecular dynamics simulation.

### 2.7. Statistics

The statistical analysis was performed using R, and data are presented as the means  ±  SDs for three independent experiments. Differences between two groups (treated and untreated cells) were assessed by the paired *t*-test. *p* < 0.05 was considered statistically significant. Regarding the methylation difference calculation, the fast Fisher test was performed. *q*-value < 0.01 indicated a statistically significant difference.

## 3. Results

### 3.1. IND Induces the Expression of LC3-II and Beclin1 in Caco-2 Cells

**Figure 1:**
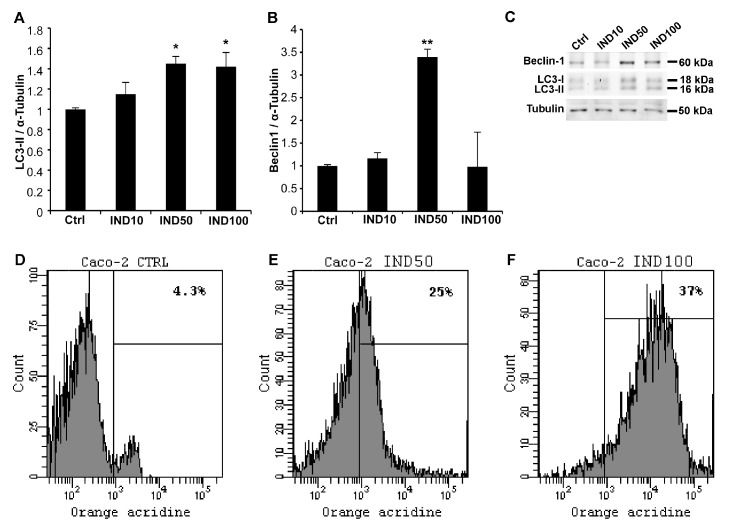
Indicaxhanthin (IND) induces autophagy in Caco-2 cells. LC3-II (**A**) and Beclin1 (**B**) expression in treated and untreated Caco-2 cells (mean ± SD). Graphic representation (average of three independent experiments) of band densitometric analyses performed using ImageJ software. Data were normalized to α-tubulin. Asterisks represent the *p*-value: * *p* <  0.05, ** *p* < 0.01. (**C**) Representative Western blotting assay. (**D**–**F**). Representative plots for acidic vesicular organelles’ quantitation in Caco-2 cells by flow cytometry. Caco-2 cells were exposed to the medium either unsupplemented (**D**) or supplemented with IND50 (**E**) or IND100 (**F**). After 48 h, the cells were collected, stained with 100 μg/mL acridine orange, and subsequently analysed in a flow cytometer. The analyses were performed in triplicate.

To evaluate the potential effect of IND in inducing autophagy, the expression levels of two autophagic markers, LC3-II and Beclin1, were evaluated by Western blot analysis. The results indicate that the expression of the LC3-II protein increased in a dose-dependent manner in the IND-treated Caco-2 cells compared with the untreated control (Figure 1A). Exposure to IND50 induced the up-regulation of Beclin1 by about 3.5-fold (Figure 1B).

### 3.2. IND Increases Accumulation of Acidic Vesicular Organelles in Caco-2 Cells

To further evaluate the effects of IND on the autophagic process, the quantification of acidic vesicular organelles by flow cytometry was performed (Figure 1D–F). Caco-2 cells treated with IND50 and IND100 showed substantial increases in red fluorescence intensity by about 6- and 9-fold, respectively, compared with the untreated cells, indicating autophagolysosome production. As expected from the expression of the autophagic markers, IND10 failed to induce significant variations in fluorescence intensity with respect to the controls.

### 3.3. IND Modulates Global CpG Methylation and Affects the Methylation of Autophagic Genes

#### 3.3.1. Global Analysis of RRBS Data

We previously reported that IND inhibited DNMT activity and induced the epigenetic modulation of silenced oncosuppressor genes in Caco-2 cells [23]. To evaluate if the observed pro-autophagic effect of IND involved the epigenetic modulation of autophagic genes, methylomic studies were performed.

**Figure 2:**
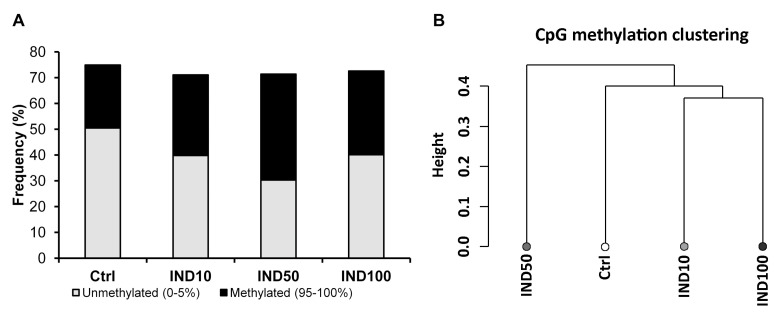
Reduced representation bisulphite sequencing (RRBS) results. (**A**) Staked column chart of % CpG methylation. Only the fully methylated (95–100%) or unmethylated (0–5%) fractions are represented. (**B**) Hierarchical clustering graph of global genomic methylation assessed in Caco-2 cells either untreated or treated with different concentrations of IND. Distance method: correlation; clustering method: ward. See also Figures S2 and S3.

RRBS data analysis confirmed the expected bimodal profile of the methylation rate, where most CpGs displayed either high or low methylation states. The untreated Caco-2 cells showed about 50% demethylated CpG sites and only about 25% strongly methylated sites (Figure 2A). The cell treatments with IND showed a methylating effect with maximum activity at the IND50 dose, as also confirmed by the hierarchical-clustering dendrogram (Figure 2B), the principal component analysis (PCA), and Pearson correlation analyses based on the similarity of the methylation profiles of the samples (Figures S1–S3).

#### 3.3.2. Differentially Methylated Cytosine (DMC) Analysis

Considering the four phases of the autophagic process, we selected 60 promoters of 51 phase-specific genes (for nine genes, two TSSs were considered; see Table S1) and obtained results for 52 promoters of 47 phase-specific genes. The weighted averages of the variations between the methylation data of these gene promoters and those of the control for each single treatment are listed in Tables S3–S6. The analysed genes were grouped based on their role in the autophagic process.

Clustering analyses were carried out based on the methylation difference averages (Figure S4 shows the heatmap obtained) and genes showing a similar behaviour were grouped in the “Homogenous behaviour gene cluster (HoBGeC)”.

To visually represent the changes in the mean methylation levels of the promoters of the clustered genes, a bar plot was then constructed for each HobGeC.

**Figure 3:**
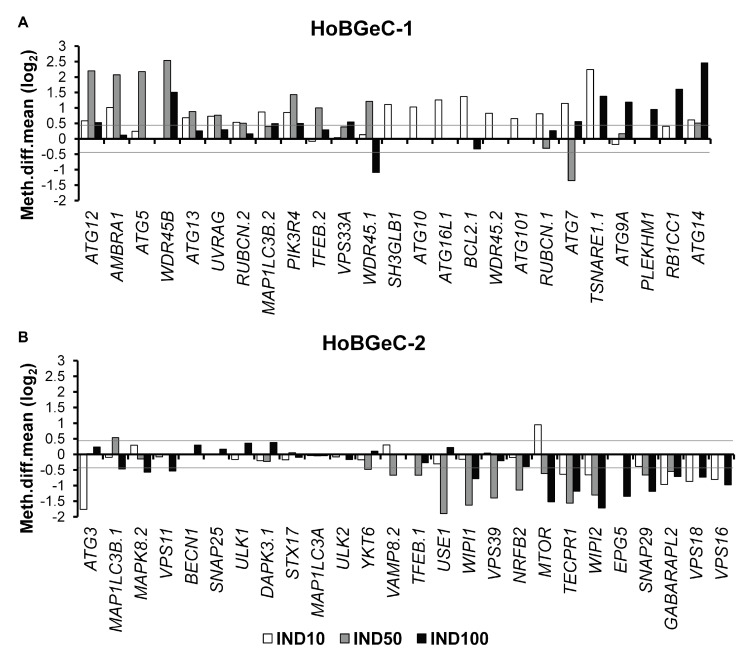
IND effects on promoter methylation of autophagy genes: DMC analysis. Representative bar plots of HoBGeC-1 (**A**) and HoBGeC-2 (**B**) weighted average values. The lines indicate a robust methylation difference with respect to the controls (corresponding to a |diff.meth| > 25%).

The treatment with IND revealed a methylating effect in at least one treatment in all the 25 genes belonging to HoBGeC-1, (Figure 3A). *ATG12*, *AMBRA1*, *ATG5*, *WDR45B*, *TSNARE1.1*, and *ATG14* were the gene promoters exhibiting a more evident methylating effect.

Regarding the HobGeC-2 group, IND produced a demethylating effect in at least one treatment on 19 of the 26 gene promoters considered (Figure 3B). *ATG3*, *USE1*, *WIPI1*, *VPS39*, *NRFB2*, *TECPR1*, *WIPI2*, *EPG5*, and *SNAP29* were the more demethylated gene promoters.

Heterogeneity was instead found for at least three genes belonging to both HobGeCs with regard to the treatments with different concentrations of IND with both methylation and demethylation phenomena (Figure 3A,B).

The effect of IND on the methylation of the MTOR promoter (mechanistic target of rapamycin (serine/threonine kinase), a major negative regulator of autophagy), is intriguing. In fact, IND10 exposure induced MTOR promoter methylation, while the IND50 and IND100 treatments induced MTOR promoter demethylation, compared with control cells.

IND50 had a methylating effect on the promoter of *WDR45.1*, while IND100 had a demethylating effect. On the contrary, IND50 had a demethylating effect on the promoter of *ATG7*, while IND10 and IND100 had methylating effects. *MTOR*, *WDR45.1,* and *ATG7* showed the presence of a CpG island in the differently methylated region. Of note, an inspection of the epigenetic functional markers of these promoters in untreated Caco-2 cells (DNAseI hypersensitivity, H3K27 acetylation, and H3K4 tri-methylation) showed that all of them exhibited active promoters (from ENCODE Consortium: Reference Caco-2 epigenome ENCSR838VOB: https://www.encodeproject.org/reference-epigenomes/ENCSR838VOB/, accessed on 30 January 2023, and Mint-ChIP-seq in Caco-2: https://www.encodeproject.org/experiments/ENCSR571QQB/, accessed on 30 January 2023). Therefore, it is conceivable that the methylating effect could inhibit transcription and could be more effective than the demethylating effect.

Considering the four phases of the autophagy process, our data show that IND induced promoter methylation changes specifically in the genes involved in the late stages, while the demethylating effect was mainly observed for the genes involved in the fusion stage (Tables S3–S6).

#### 3.3.3. Differentially Methylated Region (DMR) Analysis

To highlight the DMRs, a tile-based analysis was also performed in both the promoter regions and the gene bodies. The values with |diff.meth| > 15% at a *q*-value of <0.01 obtained for each of the three pairwise comparisons on the promoter regions and on the gene bodies are listed in Tables S7 and S8, respectively.

In Table 1, genes with a robust DMR in the promoter (with |diff.meth| > 25% at *q*-value < 0.01), the genomic coordinates of the tile, the TSS, the difference methylation values, and, if present, the annotated regulatory regions (candidate cis-regulatory elements from ENCODE or regulatory elements from “Open Regulatory Annotation” records) are listed [49,50].

According to the DMC analyses, the tile-based analyses of the promoter regions showed the demethylation of a control element located in the *ATG3* upstream region. Moreover, after treatment with IND10 and IND50, a strongly demethylated region was observed in the *BCL2.2* gene (transcript variant beta, NM_000657) promoter.

Tile analysis highlighted a previously undetected methylation after treatment with IND with respect to the controls for *ULK2*, *EPG5,* and *VPS11*, and confirmed the methylation of *MTOR* and *PIK3R4*. In the *BECN1* “promoter”, a particularly methylated tile was found downstream of the CpG island. In this region, however, no regulatory elements were annotated.

In Table 2, genes with a robust DMR in the gene body (with |diff.meth| > 25% at *q*-value < 0.01), the genomic coordinates of the tile, the difference methylation values, the annotated regulatory regions (candidate cis-regulatory elements from ENCODE or regulatory elements from “Open Regulatory Annotation” records), and the functional classification are listed.

Regarding the tile-based analysis of the gene body regions, the highest methylation difference (hypermethylation) was detected in the *PIK3R4* gene body, a region with typical features of a distal enhancer. Strong hypermethylation was found also in the *TSNARE1* gene body corresponding to an intron or an exon, depending on alternative splicing, and in the *ATG9A* gene. On the contrary, strong demethylation was found in two regulatory regions located in introns. The first was in the *GABARAPL2* gene and the second in the *ATG7* gene.

### 3.4. IND Induces BECN1 Promoter Demethylation

Considering the importance of Beclin1 in the autophagic process, its increase after cell exposure to IND (Figure 1B) and the unexpected up-regulation of the DNA methylation of a region downstream of the TSS and out of the BECN1 CpG island, we further analysed the methylation levels of four CpG sites present in the CpG island of the *BECN1* promoter by MSRE-PCR.

**Figure 4:**
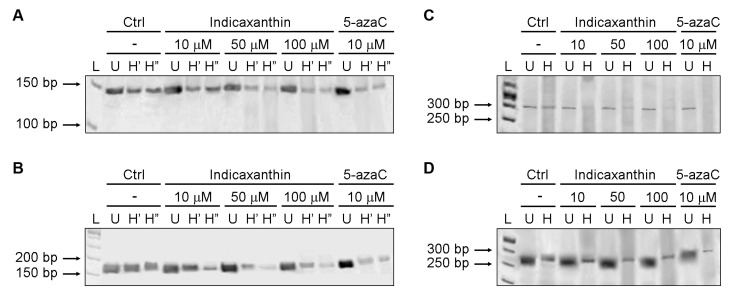
IND induces BECN1 promoter demethylation. MSRE-PCR of *BECN1* promoter in treated and untreated Caco-2 cells. Caco-2 cells were exposed to the medium, either unsupplemented (Ctrl), or supplemented with IND10, IND50, or IND100, or 10 μM 5-azaC. At the end of the incubation period, the genomic DNA was isolated and quantified, and MSRE-PCR was performed, as reported in the methods. (**A**,**B**) CpG sites 126 and 255, respectively (L = 50 bp ladder; U = undigested; H′ and H″ = samples digested by *Hpa*II). (**C**,**D**): CpG sites 299 and 472, respectively (L = 50 bp ladder; U = undigested; H = samples digested by *Hha*I).

The results of the MSRE-PCR experiments are reported in Figure 4. For 126 (Figure 4A) and 255 sites (Figure 4B), the decrease in band intensity from the undigested samples to the digested samples was more robust for the DNA obtained from Caco-2 cells treated with IND50 and IND100 than for that from the untreated and IND10-treated cells. The observed decrease was weaker for the 126 site than that for the 255 site. For the 299 site (Figure 4C), the band intensity of the undigested sample was similar to that of the digested sample from untreated cells, suggesting high methylation levels. On the contrary, for this site, a weaker band was also observed in the digested samples from IND-treated cells when compared with the corresponding undigested samples. Of note, a more evident variation in intensity was shown in the samples treated with IND100.

For the 472 site (Figure 4D), the decrease in the band intensity from the undigested samples to the digested ones was weaker in cells treated with IND than that in untreated cells. Collectively, the obtained results suggest that IND can methylate the 472 site and demethylate the other analysed sites, showing a stronger effect on the 255 and 299 sites. As expected, 5-azaC caused the demethylation of the observed sites, as demonstrated by the strong weakening of the band intensity from the undigested to the digested samples.

### 3.5. IND Competes with Bcl-2-Beclin1

**Figure 5:**
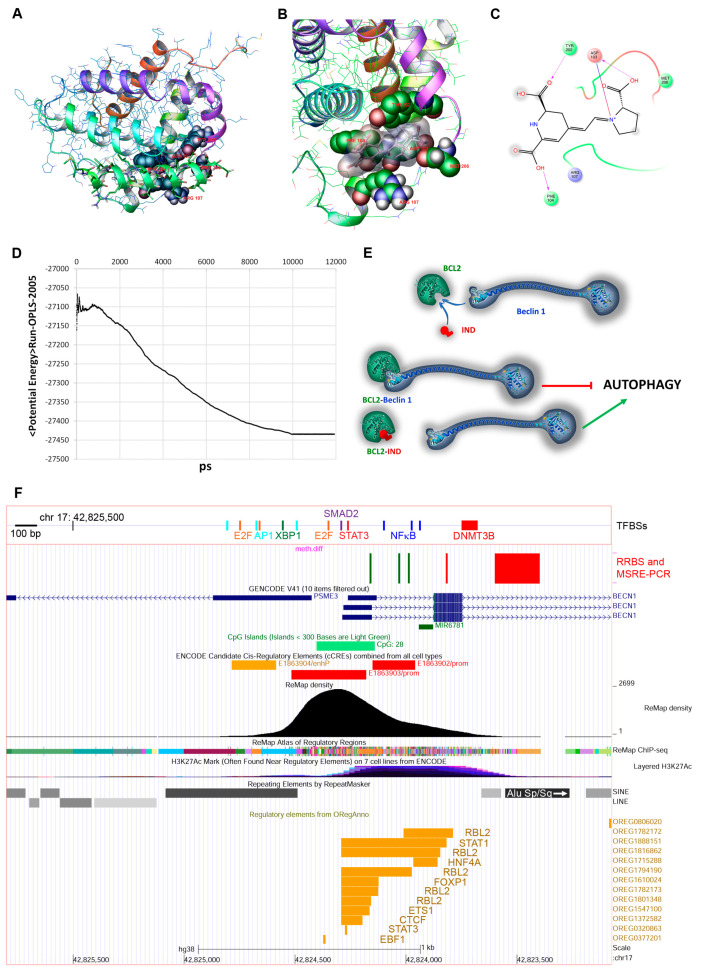
IND affects Beclin1−Bcl-2 interaction and BECN1 regulation. (**A**) Three-dimensional representation of binding of Beclin1 to Bcl-2 active site (PDB id 5VAU). (**B**) Three-dimensional representation and (**C**) amino acids map of the IND − Bcl-2 binding mode after 10 ns of molecular dynamic simulation. (**D**) Potential energy in the molecular dynamic simulation for the IND–Bcl-2 complex. (**E**) Hypothetical interference mechanism of IND in Beclin1−Bcl-2 interaction. Protein drawings are based on Bcl-2 (PDB ID 5VAU) and Beclin1 (Alphafold ID AF-Q14457-F1) structures. (**F**) UCSC Genome Browser view of the *BECN1* promoter (hg38 assembly). The Browser view includes a custom track, which shows RRBS and MSRE-PCR results. Top: known transcription factors and DNMT3B binding sites.

To gain more insight into the potential binding capability of IND to the Beclin1 binding site of Bcl-2, molecular modelling studies were performed. A model of the IND-Bcl-2 complex, based on guided IFD simulations starting from the crystal structure of the Bcl-2-Beclin1 complex (Figure 5A), was obtained. The best-scored IFD output was submitted to 20 ns of molecular dynamics simulation to assess the complex stability of IND and to analyse the amino acids involved in the interaction. Figure 5D shows that the complex reached reasonable stability around 10 ns, and further analysis of the amino acids involved in the binding (Figure 5B,C) confirmed the capability of IND to bind to the active site.

The molecular dynamics simulation of the Bcl-2-IND complex showed that the amino acids of Bcl-2 close to IND were also involved in the binding of Bcl-2 to Beclin1 (Figure 5A–C). In fact, TYR202, ASP103, MET206, ARG107, and PHE104 residues seemed to be important in the interaction between both Bcl-2 and Beclin1, and Bcl-2 and IND.

## 4. Discussion

Autophagy is an evolutionarily conserved process by which malfunctioning or senescent cellular constituents are sequestered in autophagolysosomes, then degraded and recycled. This process is crucial for maintaining homeostasis by preserving genome stability [51] and contributing to the response to oxidative stress [52]. On the other hand, evidence suggests an important role of autophagy in the physiopathology of many diseases, first of all, the intestinal bowel diseases. Of note, a dichotomic role was shown in cancer [53]. Recently, the potential of autophagy inducers in cancer prevention or treatment was evaluated. Several synthetic or natural molecules, including resveratrol [54], curcumin [55], and quercetin [56], were reported to induce in vitro autophagy in different cancer cell lines. Although the exact mechanisms are still not clear, it was demonstrated that some autophagy inducers might interfere with the PI_3_K/AKT/mTOR pathway, AMPK activity, and Bcl-2-Beclin1 complex formation. However, the involvement of epigenetic mechanisms in pro-autophagic effects has not yet been investigated.

Among phytochemicals, IND, a betalain pigment present in prickly pear fruit, has been extensively studied for its antioxidant, anti-inflammatory, and antiproliferative activities [23,24]. The biological activity of IND, as that of other natural antioxidants, implies redox-active proprieties. Owing to their ability to influence the cellular redox state, antioxidant molecules not only protect cells from oxidative stress phenomena, but can also induce changes in the function of biological targets with redox-sensitive sites [57]. Additionally, a direct interaction of some phytochemicals with specific biological targets in cells was demonstrated, affecting their function [58]. In particular, it was shown that IND inhibited the activity of DNMTs in vitro, and in silico molecular modelling studies suggestied that this inhibition involved its binding to the catalytic site of the DNMT1 enzyme. Nevertheless, *DNMT3A* expression was upregulated [23]. We previously showed that the IND promoted the inhibition of the proliferation of human colon cancer Caco-2 cells, which was associated with the epigenetic modulation of some methylation-silenced oncosuppressor genes, including *p16^INK4a^* [23,24]. However, no studies have investigated the epigenetic potential of IND in the methylation status of the autophagy genes and its ability to act as an autophagy inducer. In the present study, we also evaluated whether IND played a role in the induction of autophagy in Caco-2 cells and if epigenetic mechanisms were involved. This study, by contributing to the definition of the gut cell-specific epigenomic profile of IND phytochemical, can be of nutritional interest: it provides data to better understand IND’s peculiar epigenetic modulation power, even to consider it as a dietary adjuvant of traditional drug therapies of intestinal pathologies.

The IND concentrations selected for our experiments are compatible with the phytochemical concentrations that can be obtained at the luminal level after ingesting a quantity of yellow cactus pear fruit between 30 g and 300 g [20].

In addition, we previously reported that the treatment of proliferating Caco-2 cells with IND for 48 h caused a concentration-dependent inhibition of cell growth with an IC_50_ of 115 µM [24].

Here, under the same experimental conditions, the pro-autophagic potential of IND was demonstrated by the up-regulation of the autophagic markers LC3-II and Beclin1, and the increased production of autophagolysosomes. Beclin 1 protein expression was not correlated with the indicaxanthin concentrations used in our study: in fact, the Beclin 1 protein levels peaked at 50 µM and decreased when 100 µM of IND was used. This unexpected result might be attributed to a reduced ability of cells to respond to the phytochemical when its concentration is close to IC_50_. It is possible that, at 100 µM, indicaxanthin might represent a stress exceeding the threshold of a well-controlled cellular response. The trend of Beclin 1 expression could be the result of a hormetic effect of IND, similar to what has been observed for many phytochemicals and redox-active molecules.

LC3s (microtubule-associated protein 1 light chain 3), encoded by a gene family, an orthologue of ATG8 in yeast, contains three members in humans: *MAP1LC3A*, *MAP1LC3B,* and *MAP1LC3C*, which are proteins ubiquitously distributed in mammalian tissues [59]. LC3-I is a soluble protein. During autophagy, LC3-I is lipidated with the phosphatidylethanolamine of the autophagosomal membranes by an autophagy-related ubiquitylation-like conjugation system (E1-like ATG7 and E2-like ATG3) to form the LC3–phosphatidylethanolamine conjugate named LC3-II. Thus, detecting LC3-II is a useful method of monitoring autophagy [60].

Beclin1 is required for the formation of PI3KC3-C2 (class III phosphatidylinositol 3-kinase complexes-C2), and its activity is regulated by its interaction with several proteins at the post-translational level. The best-known interactors are the Bcl-2-like proteins. The dissociation of Beclin1 from Bcl-2 induces autophagy; therefore, the regulation of this association is crucial. Different mechanisms are involved in the dissociation of Beclin1 and Bcl-2/Bcl-XL during autophagy: post-translational modifications, the competitive displacement of Bcl-2 by other Beclin1-binding proteins, or the competitive displacement of Beclin1 by other Bcl-2 interactors [61]. Despite the limitations of the method, our in silico molecular modelling data suggested that IND could bind directly to Bcl-2. More specifically, by comparing the complexes Bcl-2–Beclin1 and Bcl-2–IND, we found that the same amino acids of Bcl-2 involved in the binding with Beclin1 were equally involved in the Bcl-2–IND interaction. Therefore, competition between Beclin1 and IND for the same Bcl-2 sites might be conceivable (Figure 5E). This result would indicate that IND could modulate Beclin1 acting on both protein levels and activity.

By methylomic approaches, we showed that epigenetic mechanisms are also involved in the pro-autophagic activity of IND.

Although specific regions, such as some tumour-suppressor genes, are often found to be hypermethylated, it is well known that global DNA hypomethylation is a hallmark of cancer cells [62]. Caco-2 cells show low global levels of DNA methylation (only about 25% strongly methylated sites). Cell exposure to IND promoted a genomic CpG hypermethylation and IND50 showed the most pronounced effect. In addition, the elaboration of the RRBS data of the autophagy gene promoters performed by analysing both DMC (single CpGs) and DMR (tiles) highlighted the existence of gene-specific epigenetic effects. In particular, 26 out of 47 genes showed methylation levels markedly higher than those of the controls. In contrast, IND produced a robust demethylating effect on 13 of the observed genes. These differences are indicative of the gene-specific actions of the phytochemical. We previously showed that IND not only modulated DNMT protein levels, but also affected the expression of some genes encoding enzymes involved in DNA demethylation, such as TET2 and MBD4 [23]. These, together with the already-demonstrated ability to influence the DNMT enzyme activity, could account for the observed gene specificity of the phytochemical.

In the context of the autophagic process, these data indicate that IND influenced the methylation level, especially that of those genes that regulate the late stages of autophagy, such as autophagosome fusion and elongation.

Among those genes, *ATG7*, *ATG3*, and *ATG10* encode proteins essential for mammalian autophagy, and are involved in two ubiquitylation-like modifications of target proteins, ATG12-conjugation and LC3-modification. E1-like ATG7, E2-like ATG10, and E3-like ATG5 are involved in the ATG12 conjugation that is essential for the formation of pre-autophagosomes (or phagophores). E1-like ATG7 and E2-like ATG3, whose gene promoters are demethylated by IND, are involved in LC3 lipidation, an event necessary for phagophore expansion and closure, and are also important in the E3-like ATG12–ATG5–ATG16L1 complex formation [63,64].

Among those genes affected by IND exposure, *WIPI1* and *WIPI2* encode WD40 domain-containing proteins. WIPI1 and WIPI2 (WD repeat-domain phosphoinositide-interacting protein 1 and 2) recruit the E3-like ATG12–ATG5–ATG16L1 complex that directly controls the elongation of the nascent autophagosomal membrane to phagophore assembly sites [65].

ATG12–ATG5 also binds TECPR1 (Tectonin beta-propeller repeat-containing protein 1), which promotes the fusion of LC3C autophagosomes with lysosomes [66]. This process is coordinated by the SNARE proteins (soluble N-ethylmaleimide-sensitive factor-attachment protein receptors), including USE1 (vesicle-transport protein USE1), SNAP29 (Synaptosomal-associated protein 29), and TSNARE1 (t-SNARE domain-containing protein 1) [67].

We also found an epigenetic effect of IND on genes involved in the early signalling events of autophagy. Among these, *BCL2* is a gene involved in the nucleation phase, whose expression is regulated by two promoters [68,69]. RRBS analysis showed a methylating effect of IND at the lowest concentration in the region corresponding to the first promoter. Differently, DMR analysis highlighted a hypomethylated tile after treatment with IND10 and IND50 for the second promoter. This region, located on the P2 promoter, was previously also identified as a regulatory element for the P1 promoter [70]. Putatively, this tile binds the VDR (vitamin D3 receptor), SMARCA4 (transcription activator BRG1, component of SWI/SNF-related chromatin remodelling complexes, and component of the CREST–BRG1 complex), and EGR1 (early growth-response protein 1) transcription factors (OREG1937747, OREG1250101, and OREG0396743, respectively). It has also been shown that the “pioneer” factor Foxa1 (Forkhead box A1/Hepatocyte nuclear factor 3-alpha) decreased the transcription activity of the *BCL2* promoter under normal conditions and oxidative stress in the A549 cell line [71].

Moreover, data analysis performed on gene bodies allowed us to discover regions not belonging to the promoter, which are diversified by IND treatments in terms of DNA methylation. The gene body may contain regulatory regions different from those of the promoter that can influence promoter activity by binding transcription factors. Thus, we highlighted other gene regions that are differently methylated.

Strongly hypermethylated tiles were found, for example, in the *PIK3R4* and *TSNARE1* gene bodies; moreover, hypermethylated tiles were also found in *WDR45*, *EPG5*, and *ATG9A* genes. The hypermethylated tile in the *ATG9A* gene body contained a putative binding site for EGR1, like the *BCL2* gene promoter (demethylated by IND10 treatment), *MAPK8.2* promoter (unaffected), and also the *SNAP29* gene body (slightly demethylated).

On the other hand, hypomethylated tiles, annotated as distal enhancers, were found in the *ATG7* and *GABARAPL2* gene bodies. However, these signatures are not present in untreated Caco-2 cells. In particular, the tile in the *ATG7* gene could bind SMARCA4, DUX4 (double homeobox 4, usually not expressed in adult cells), JUN (Jun proto-oncogene, AP-1 transcription factor subunit), and GATA2 (OREG1256740, OREG0563863, OREG0730235, and OREG1675345, respectively). This region is particularly interesting because it has different chromatin states (and, therefore, different histone post-translational modifications) in different cell types, having signatures ranging from quiescent to strongly transcribed features, or from enhancer to active promoter features in different cell types (data from the Roadmap Epigenomic project at https://egg2.wustl.edu/roadmap/web_portal/chr_state_learning.html#core_15state, accessed on 30 January 2023).

Regarding the methylation status of the promoter of the *BECN1* gene encoding Beclin1, we showed that at least two regulatory regions displayed changes in DNA methylation in Caco-2 cells after IND treatments: a tile in the second intron (detected by RRBS) that was more methylated, and at least two CpG dinucleotides in the first intron (detected by MSRE-PCR) that were less methylated (Figure 5F). Such epigenetic variations could affect *BECN1* transcription and contribute to the observed changes in Beclin1 expression. In a similar fashion, it was demonstrated that DNMT3B bonded to the *BECN1* promoter, causing an increase in DNA methylation and a decrease in protein expression in tamoxifen-resistant breast cancer (MCF7/TAMR) cells. Moreover, H19 lncRNA knockdown promoted the interaction between DNA methyltransferase and the *BECN1* promoter [72]. Regarding the regulatory region of *BECN1*, the tile that was more methylated was located in the region between the positions +700 and +900 bp with respect to the TSS. Moreover, its sequence showed 94% identity with the left monomer of an Alu element (S subfamily), a primate-specific repetitive retrotransposon. Although no functional data are available for this sequence (except for the enhancer signatures highlighted in the roadmap epigenomics database), it is known that transposable elements can have profound effects on genome structure and function and in gene regulation [73]. Alu elements contain a number of potential transcription factor binding sites and can show enhancer and/or silencer activities [74]. For example, a cis-regulatory element of the *CD8A* gene is composed of an Alu repeat and exhibits both enhancer and silencer activities [75].

However, the regulation of *BECN1* transcription mainly depended on a different region, located between −1000 and +500 bp with respect to the TSS, where a variety of transcription factors can bind and drive *BECN1* expression. Some of them act as activators, such as NFκB, HIF1α, c-Jun/Fos, E2F1, and XBP1, and others as repressors, like Smad2 and STAT3 [76,77,78,79,80,81,82,83]. In particular, the CpGs showing a decrease in methylation after IND treatment were near the NFκB binding sites (Figure 5F). It was demonstrated that the *BECN1* promoter activity increased remarkably when *RELA*, encoding RelA/p65, a key subunit of NFκB, was overexpressed [84,85].

Overall, our results show that IND promotes autophagy in intestinal epithelial cancer cells at similar concentrations to the luminal ones after prickly pear fruit consumption. Our results show that the increased expression of autophagy markers is associated with differential DNA methylation, not only in the promoters, but also in the enhancers of the genes. However, other studies are needed to demonstrate the importance of the epigenetic regulatory outcome in the pro-autophagic effects of IND. Finally, molecular modelling data suggest that IND could directly affect autophagy regulators’ activities.

Further research can complete the data described and reported here. In particular, it will be interesting to study the global effect of IND on the methylome of Caco-2 cells, highlighting other possible pathways involved in the cell response to IND exposure and completing its nutrigenomic profile. These data can contribute to the characterization of the potential beneficial effects of this dietary phytochemical in the gastrointestinal tract, shedding light on the relationship between diet, IND nutrigenomic effects, and intestinal bowel diseases.

## Figures and Tables

**Table 1:** Tile analysis results: differentially methylated tiles (200 bp-diff.meth%) found in the “promoter” regions of selected genes. The last column shows the corresponding annotated regulatory region. cCRE: ENCODE candidate cis-regulatory elements. OregAnno: regulatory elements from “Open Regulatory Annotation” records. Robust DMRs (|diff.meth| > 25% at *q*-value < 0.01) are in bold. n.s.: not significant. For the complete results, see also Table S6.

Gene Symbol	Strand	Tile Chr	Tile Start	Tile End	TSS	IND10	IND50	IND100	EH38 cCRE/ORegAnno
***MTOR***	−1	chr1	11,263,201	11,263,400	11,262,551	**29.8**	n.s.	n.s.	E1318739
***ULK2***	−1	chr17	19,867,801	19,868,000	19,867,936	24.4	n.s.	**27.8**	E1851797
***BECN1***	−1	chr17	42,823,401	42,823,600	42,824,282	**26.4**	**48.9**	n.s.	intron
***PIK3R4***	−1	chr3	130,747,201	130,747,400	130,746,829	**31.4**	**36.3**	n.s.	OREG1230891
***BCL2.2***	−1	chr18	63,318,401	63,318,600	63,318,812	**−34.7**	**−51.0**	n.s.	E1923293
***ATG3***	−1	chr3	112,562,801	112,563,000	112,561,962	**−39.9**	n.s.	−19.8	E2227361/2
***EPG5***	−1	chr18	45,966,201	45,966,400	45,967,329	**33.7**	n.s.	n.s.	intron
***VPS11***	1	chr11	119,068,401	119,068,600	119,067,692	**28.2**	**30.8**	n.s.	OREG1260042 OREG1081042

**Table 2:** Tile analysis results: differentially methylated tiles (200 bp-diff.meth %) found in the gene body regions of selected genes. The last two columns show annotated regulatory regions and notes. cCRE: ENCODE candidate cis-regulatory elements. ORegAnno: regulatory elements from “Open Regulatory Annotation” records. Robust DMRs (|diff.meth| > 25% at *q*-value < 0.01) are in bold. n.s.: not significant. For the complete results, see also Table S7.

Gene Symbol	Strand	Tile Chr	Tile Start	Tile End	IND10	IND50	IND100	EH38 cCRE/ORegAnno/CpGI	Classification
***MTOR***	−1	chr1	11,122,601	11,122,800	n.s.	**−46.8**	n.s.		intron
***ULK1***	1	chr12	131,919,801	131,920,000	n.s.	n.s.	**−26.4**		intron
***ATG9A***	−1	chr2	219,219,401	219,219,600	14.6	n.s.	**50.7**	E2075977	enhP (near to ABCB6 prom)
***PIK3R4***	−1	chr3	130,727,401	130,727,600	n.s.	**73.7**	n.s.	E2238076/7	enhD
***ATG7***	1	chr3	11,379,801	11,380,000	**−36.6**	n.s.	**−28.5**	**E2178411**	enhD
***WDR45.1/.2***	−1	chrX	49,099,601	49,099,800	**29.7**	n.s.	24.0	H3K27Ac in K562	intron
***EPG5***	−1	chr18	45,966,201	45,966,400	**33.7**	n.s.	n.s.		intron
***GABARAPL2***	1	chr16	75,571,801	75,572,000	n.s.	−17.6	**−38.4**	E1828346	intron
***TSNARE1***	−1	chr8	142,249,201	142,249,400	n.s.	**−26.5**	**n.s.**		intron
		chr8	142,261,601	142,261,800	**36.9**	n.s.	n.s.	OREG1946662	intron
		chr8	142,271,201	142,271,400	**61.1**	**62.6**	**27.8**	OREG1517008	exon or intron
		chr8	142,375,201	142,375,400	n.s.	n.s.	**35.8**		intron

## Data Availability

The data presented in the current study are available from the corresponding author upon request.

## Supplementary Materials

The following supporting information can be downloaded at: https://www.mdpi.com/article/10.3390/nu15153495/s1. Table S1: Complete list of selected genes and chromosomal ranges corresponding to their promoters and gene bodies; Table S2: Oligonucleotides used in this study, related to the MSRE-PCR method; Figures S1–S3: RRBS datasets’ statistical analyses; Figure S1: Pearson correlation coefficients calculated using the *methylKit* R package: IND10, IND50, and IND100 RRBS datasets; Figure S2: RRBS datasets—principal component analysis (PCA) calculated using the *methylKit* R package: scree plot; Figure S3: RRBS datasets—PCA calculated using the *methylKit* R package: scatter plot; Table S3: Averaged differential methylation data (log2 weighted average values) of each CpG (min.cov = 10) located in the promoter region of genes involved in autophagy induction; Table S4: Averaged differential methylation data (log2 weighted average values) of each CpG (min.cov = 10) located in the promoter region of genes involved in the phagophore nucleation process; Table S5: Averaged differential methylation data (log2 weighted average values) of each CpG (min.cov = 10) located in the promoter region of genes involved in the phagophore elongation process; Table S6: Averaged differential methylation data (log2 weighted average values) of each CpG (min.cov = 10) located in the promoter region of genes involved in the fusion process; Figure S4: Heatmap obtained using the average values of the methylation difference of the studied promoters. PIK3C3, RAB7A, VPS41, and INPP5E genes were excluded due to the absence of significant data; Table S7: Promoter DMRs (tiles) and their features, related to Table 1; Table S8: Gene body DMRs (tiles) and their features, related to Table 2. References [49,50] are cited in supplementary materials.
