# Supplementary material for: Indicaxanthin Induces Autophagy in Intestinal Epithelial Cancer Cells by Epigenetic Mechanisms Involving DNA Methylation
Source: Nutrients. 2023 Aug 7;15(15):3495. doi: 10.3390/nu15153495 (PMC10420994; doi:10.3390/nu15153495)
Supplement: Supplementary file 1 [file nutrients-15-03495-s001.zip › nutrients-2517549-supplementary.pdf]

**Indicaxanthin induces autophagy in intestinal epithelial cancer cells by epigenetic mechanisms involving DNA methylation**

**Nutrients**

Maria Antonietta Ragusa, Ilenia Cruciata, Chiara La Rosa, Chiara Schimmenti, Flores Naselli, Sara Volpes, Graziella Serio, Maurizio Mauro, Mariangela Librizzi, Claudio Luparello, Roberto Chiarelli, Antonino Lauria, Carla Gentile, Fabio Caradonna

Corresponding author: Carla Gentile, Università di Palermo, Dipartimento di Scienze e Tecnologie Biologiche Chimiche e Farmaceutiche (STEBICEF), Palermo, Italia. E-mail: carla.gentile@unipa.it

**Supplemental material: Tables S1-S8 and Figures S1-S4**

**Table S1:** Complete list of selected genes and chromosomal ranges corresponding to their promoters and gene bodies

| gene | strand | hg38.coordinates | chr | prom.start | prom.end | body.start | body.end |
|---|---|---|---|---|---|---|---|
| MTOR | -1 | chr1:11,106,535-11,262,551 | chr1 | 11261551 | 11263551 | 11106535 | 11261551 |
| ULK1 | 1 | chr12:131,894,622-131,923,150 | chr12 | 131893622 | 131895622 | 131895622 | 131923150 |
| ULK2 | -1 | chr17:19,770,830-19,867,936 | chr17 | 19866936 | 19868936 | 19770830 | 19866936 |
| ATG13 | 1 | chr11:46,617,577-46,674,520 | chr11 | 46616577 | 46618577 | 46618577 | 46674520 |
| ATG101 | 1 | chr12:52,069,971-52,077,494 | chr12 | 52068971 | 52070971 | 52070971 | 52077494 |
| RB1CC1 | -1 | chr8:52,622,458-52,714,435 | chr8 | 52713435 | 52715435 | 52622458 | 52713435 |
| ATG9A | -1 | chr2:219,219,380-219,229,717 | chr2 | 219228717 | 219230717 | 219219380 | 219228717 |
| BECN1 | -1 | chr17:42,810,134-42,824,282 | chr17 | 42823282 | 42825282 | 42810134 | 42823282 |
| PIK3C3 | 1 | chr18:41,955,234-42,087,830 | chr18 | 41954234 | 41956234 | 41956234 | 42087830 |
| PIK3R4 | -1 | chr3:130,678,934-130,746,829 | chr3 | 130745829 | 130747829 | 130678934 | 130745829 |
| ATG14 | -1 | chr14:55,366,392-55,411,858 | chr14 | 55410858 | 55412858 | 55366392 | 55410858 |
| AMBRA1 | -1 | chr11:46,396,414-46,591,364 | chr11 | 46592948 | 46594948 | 46396414 | 46592948 |
| UVRAG | 1 | chr11:75,815,210-76,144,232 | chr11 | 75814210 | 75816210 | 75816210 | 76144232 |
| NRFB2 | 1 | chr10:63,133,328-63,155,024 | chr10 | 63132328 | 63134328 | 63134328 | 63155024 |
| TFEB.1 | -1 | chr6:41,683,978-41,735,608 | chr6 | 41734608 | 41736608 | 41683978 | 41734608 |
| TFEB.2 | -1 | chr6:41,683,978-41,735,608 | chr6 | 41733570 | 41736782 | 41683978 | 41733570 |
| BCL2.1 | -1 | chr18:63,123,346-63,320,128 | chr18 | 63319128 | 63321128 | 63123346 | 63319128 |
| BCL2.2 | -1 | chr18:63,313,802-63,318,812 | chr18 | 63317812 | 63319812 | 63313802 | 63317812 |
| ATG3 | -1 | chr3:112,532,510-112,561,962 | chr3 | 112560962 | 112562962 | 112532510 | 112560962 |
| ATG5 | -1 | chr6:106,184,476-106,325,760 | chr6 | 106324760 | 106326760 | 106184476 | 106324760 |
| ATG7 | 1 | chr3:11,272,416-11,557,665 | chr3 | 11271416 | 11273416 | 11273416 | 11557665 |
| ATG12 | -1 | chr5:115,828,200-115,841,565 | chr5 | 115840565 | 115842565 | 115828200 | 115840565 |
| ATG16L1 | 1 | chr2:233,251,673-233,295,669 | chr2 | 233250673 | 233252673 | 233252673 | 233295669 |
| ATG10 | 1 | chr5:81,972,023-82,256,133 | chr5 | 81971023 | 81973023 | 81973023 | 82256133 |
| MAP1LC3B.1 | 1 | chr16:87,383,953-87,404,779 | chr16 | 87382953 | 87384953 | 87384953 | 87404779 |
| MAP1LC3B.2 | 1 | chr16:87,383,953-87,404,779 | chr16 | 87391745 | 87392506 | 87392506 | 87404779 |
| WIPI1 | -1 | chr17:68,421,281-68,457,496 | chr17 | 68456496 | 68458496 | 68421281 | 68456496 |
| WIPI2 | 1 | chr7:5,190,196-5,231,524 | chr7 | 5189196 | 5191196 | 5191196 | 5231524 |
| WDR45B | -1 | chr17:82,614,562-82,648,444 | chr17 | 82647444 | 82649444 | 82614562 | 82647444 |
| WDR45.1 | -1 | chrX:49,074,640-49,101,121 | chrX | 49100121 | 49102121 | 49074640 | 49100121 |
| WDR45.2 | -1 | chrX:49,074,640-49,101,121 | chrX | 49079073 | 49081073 | 49074640 | 49079073 |
| RUBCN.1 | -1 | chr3:197,668,867-197,736,904 | chr3 | 197735904 | 197737904 | 197668867 | 197735904 |
| RUBCN.2 | -1 | chr3:197,668,867-197,736,904 | chr3 | 197748699 | 197750699 | 197668867 | 197748699 |
| RAB7A | 1 | chr3:128,726,122-128,814,796 | chr3 | 128725122 | 128727122 | 128727122 | 128814796 |

| | | | | | | | |
|---|---|---|---|---|---|---|---|
| PLEKHM1 | -1 | chr17:45,435,900-45,490,721 | chr17 | 45489721 | 45491721 | 45435900 | 45489721 |
| TECPR1 | -1 | chr7:98,214,624-98,252,232 | chr7 | 98251232 | 98253232 | 98214624 | 98251232 |
| EPG5 | -1 | chr18:45,847,609-45,967,329 | chr18 | 45966329 | 45968329 | 45847609 | 45966329 |
| INPP5E | -1 | chr9:136,428,619-136,439,845 | chr9 | 136438845 | 136440845 | 136428619 | 136438845 |
| STX17 | 1 | chr9:99,906,654-99,974,534 | chr9 | 99905645 | 99907645 | 99907645 | 99974534 |
| SNAP29 | 1 | chr22:20,859,007-20,891,214 | chr22 | 20858007 | 20860007 | 20860007 | 20891214 |
| VAMP8.1 | 1 | chr2:85,577,586-85,582,031 | chr2 | 85576491 | 85578491 | 85578491 | 85582031 |
| VAMP8.2 | 1 | chr2:85,577,586-85,582,031 | chr2 | 85560720 | 85562493 | 85562493 | 85582031 |
| VPS11 | 1 | chr11:119,067,692-119,081,978 | chr11 | 119066692 | 119068692 | 119068692 | 119081978 |
| VPS16 | 1 | chr20:2,840,745-2,866,732 | chr20 | 2839745 | 2841745 | 2841745 | 2866732 |
| VPS18 | 1 | chr15:40,894,450-40,903,975 | chr15 | 40893450 | 40895450 | 40895450 | 40903975 |
| VPS33A | -1 | chr12:122,229,564-122,266,494 | chr12 | 122265494 | 122267494 | 122229564 | 122265494 |
| VPS39 | -1 | chr15:42,158,701-42,208,316 | chr15 | 42207316 | 42209316 | 42158701 | 42207316 |
| VPS41 | -1 | chr7:38,722,974-38,909,191 | chr | 38908191 | 38910191 | 38722974 | 38908191 |
| SH3GLB1 | 1 | chr1:86,704,576-86,748,184 | chr1 | 86703576 | 86705576 | 86705576 | 86748184 |
| MAPK8.1 | 1 | chr10:48,401,648-48,439,360 | chr10 | 48400612 | 48402612 | 48402612 | 48439360 |
| MAPK8.2 | 1 | chr10:48,401,648-48,439,360 | chr10 | 48305743 | 48307743 | 48307743 | 48439360 |
| DAPK3.1 | -1 | chr19:3,958,453-3,971,099 | chr19 | 3970099 | 3972099 | 3958453 | 3970099 |
| DAPK3.2 | -1 | chr19:3,958,453-3,971,099 | chr19 | 3968828 | 3970828 | 3958453 | 3968828 |
| GABARAPL2 | 1 | chr16:75,566,379-75,577,881 | chr16 | 75565379 | 75567379 | 75567379 | 75577881 |
| MAP1LC3A | 1 | chr20:34,558,718-34,560,345 | chr20 | 34557718 | 34559718 | 34559718 | 34560345 |
| TSNARE1.1 | -1 | chr8:142,212,080-142,403,182 | chr8 | 142402182 | 142404182 | 142212080 | 142402182 |
| TSNARE1.2 | -1 | chr8:142,212,080-142,403,182 | chr8 | 142353805 | 142355805 | 142212080 | 142353805 |
| YKT6 | 1 | chr7:44,200,978-44,214,294 | chr7 | 44199978 | 44201978 | 44201978 | 44214294 |
| USE1 | 1 | chr19:17,215,357-17,219,829 | chr19 | 17214357 | 17216357 | 17216357 | 17219829 |
| SNAP25 | 1 | chr20:10,218,830-10,307,418 | chr20 | 10217830 | 10219830 | 10219830 | 10307418 |

**Table S2.** Oligonucleotides used in this study, related to MSRE-PCR method.

| ***BECN1* CpG sites** | **Primer sequences** | **Expected fragment size (bp)** |
|---|---|---|
| *Hpa*II/*Msp*I-126 | Forward: 5'-GACTTGGGTGACGGGCTCCG-3'<br>Reverse: 5'-ACAGCCTGAGCAAAGGCAGTTTAGAGC-3' | 145 |
| *Hpa*II/*Msp*I-255 | Forward: 5'-TCCCAGGTGAAGAGCATCG-3'<br>Reverse: 5'-GAGGGAAGTCCCAACCTGCG-3' | 189 |
| *Hha*I-299 | Forward: 5'-CCGAGGCCACAGTGCCTCCG-3'<br>Reverse: 5'-AAGCGACGCCCTTGACCTCCG-3' | 302 |
| *Hha*I-472 | Forward: 5'-GGGACTTCCCTCCCTTTACCATC-3'<br>Reverse: 5'-ACCTTCCACATTCTTGACCACCC-3' | 282 |

Primers used for amplification of *BECN1* promoter CpG sites. Genome coordinates of CpG analysed: 472=42,823,819/20; 299=42,823,991/2; 255=42,824,036/7; 126=42,824,165/6

**Figures S1-S3.** RRBS datasets statistical analysis:

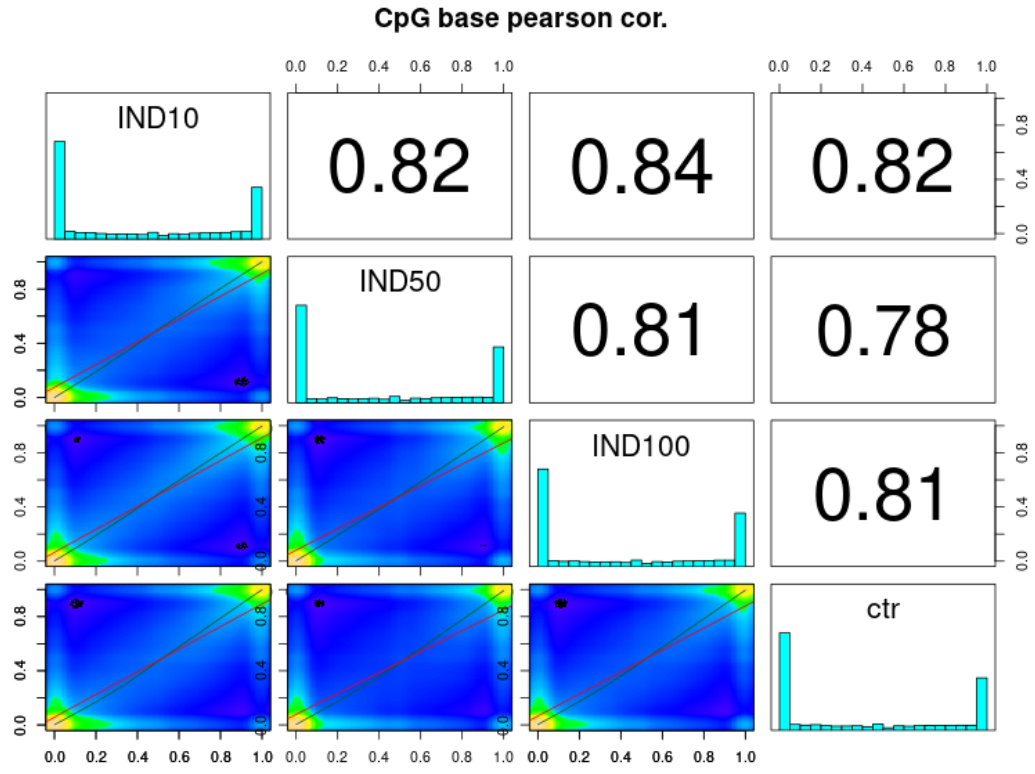

**Figure S1.** Pearson Correlation Coefficients calculated using the *methylKit* R package: IND10, IND50, and IND100 RRBS datasets.

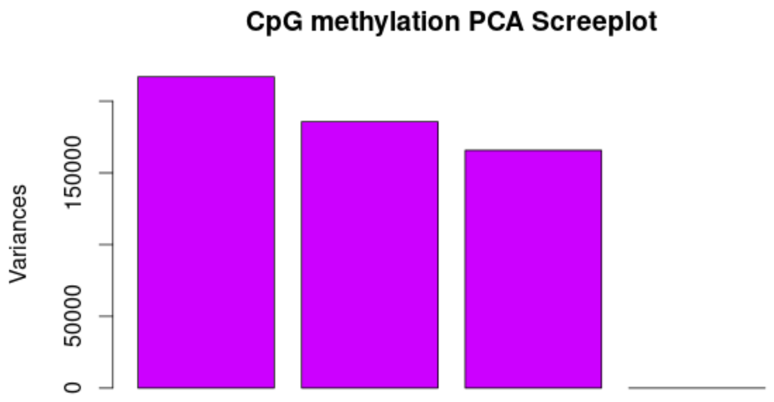


**Figure S2.** RRBS datasets - Principal Component Analysis (PCA) calculated using the *methylKit* R package: scree plot.

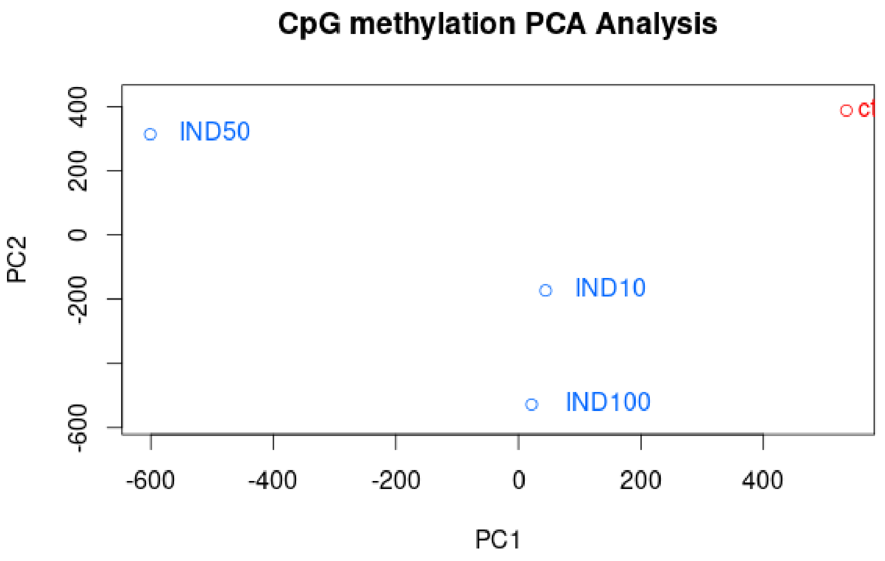


**Figure S3.** RRBS datasets – PCA calculated using the *methylKit* R package: scatter plot.

**Table S3.** Averaged differential methylation data (log2 weighted average values) of each CpG (min.cov=10) located in the promoter region of genes involved in the autophagy induction.

| INDUCTION group | IND10 | IND50 | IND100 | 5-azaC |
|---|---|---|---|---|
| *MTOR* | **0.95** | **-0.61** | **-1.52** | **1.69** |
| *ULK1* | -0.16 | - | **0.36** | **-0.45** |
| *ULK2* | -0.08 | -0.01 | -0.16 | -0.02 |
| *ATG13* | **0.68** | **0.88** | **0.25** | **0.26** |
| *ATG101* | **0.65** | - | - | **1.23** |
| *RB1CC1* | **0.4** | - | **1.6** | - |

**Table S4.** Averaged differential methylation data (log2 weighted average values) of each CpG (min.cov=10) located in the promoter region of genes involved in the phagophore nucleation process.

| NUCLEATION group | IND10 | IND50 | IND100 | 5-azaC |
|---|---|---|---|---|
| *BECN1* | - | - | **0.30** | **0.29** |
| *PIK3R4* | **0.85** | **1.43** | **0.49** | **0.75** |
| *ATG14* | **0.61** | **0.51** | **2.46** | **2.83** |
| *AMBRA1* | **1.01** | **2.07** | 0.12 | **0.95** |
| *UVRAG* | **0.73** | **0.76** | **0.29** | 0.18 |
| *NRFB2* | -0.10 | **-1.14** | **-0.39** | **0.63** |
| *TFEB.1* | 0.01 | **-0.67** | **-0.27** | -0.10 |
| *TFEB.2* | -0.08 | **1.00** | **0.29** | -0.03 |
| *BCL2.1* | **1.37** | - | **-0.33** | **1.55** |
| *RUBCN.1* | **0.81** | **-0.31** | **0.26** | **0.30** |
| *RUBCN.2* | **0.53** | **0.50** | 0.16 | -0.01 |
| *SH3GLB1* | **1.11** | - | - | **1.60** |
| *MAPK8.2* | **0.29** | -0.15 | **-0.57** | **1.31** |
| *DAPK3.1* | **-0.20** | **-0.22** | **0.38** | **-0.32** |

**Table S5.** Averaged differential methylation data (log2 weighted average values) of each CpG (min.cov=10) located in the promoter region of genes involved in the phagophore elongation process.

| ELONGATION group | IND10 | IND50 | IND100 | 5-azaC |
|---|---|---|---|---|
| *ATG9A* | -0.18 | 0.16 | **1.18** | **0.27** |
| *ATG3* | **-1.76** | 0.02 | **0.24** | **0.39** |
| *ATG5* | **0.24** | **2.18** | - | **1.68** |
| *ATG7* | **1.14** | **-1.35** | **0.56** | **0.86** |
| *ATG12* | **0.58** | **2.20** | **0.53** | 0.17 |
| *ATG16L1* | **1.26** | - | - | **0.93** |
| *ATG10* | **1.03** | - | - | 0.01 |
| *MAP1LC3B.1* | -0.10 | **0.53** | **-0.47** | **0.23** |
| *MAP1LC3B.2* | **0.87** | **0.39** | **0.49** | **0.69** |
| *WIPI1* | -0.16 | **-1.62** | **-0.78** | **0.83** |
| *WIPI2* | **-0.66** | **-1.30** | **-1.72** | **-1.01** |
| *WDR45B* | - | **2.53** | **1.50** | **0.26** |
| *WDR45.1* | 0.13 | **1.21** | **-1.09** | **0.36** |
| *WDR45.2* | **0.83** | - | - | **0.99** |
| *GABARAPL2* | **-0.96** | **-0.54** | **-0.71** | **-0.90** |
| *MAP1LC3A* | -0.04 | -0.04 | -0.04 | -0.05 |

**Table S6.** Averaged differential methylation data (log2 weighted average values) of each CpG (min.cov=10) located in the promoter region of genes involved in the fusion process.

| FUSION group | IND10 | IND50 | IND100 | 5-azaC |
|---|---|---|---|---|
| *PLEKHM1* | - | - | **0.95** | **0.41** |
| *TECPR1* | **-0.64** | **-1.56** | **-1.18** | **-0.32** |
| *EPG5* | - | - | **-1.34** | **-1.39** |
| *STX17* | -0.18 | 0.05 | -0.09 | -0.12 |
| *SNAP29* | **-0.38** | **-0.66** | **-1.18** | **-0.57** |
| *VAMP8.2* | **0.30** | **-0.67** | 0.00 | **0.59** |
| *VPS11* | -0.08 | 0.00 | **-0.53** | **-0.27** |
| *VPS16* | **-0.80** | 0.00 | **-0.97** | **-0.31** |
| *VPS18* | **-0.87** | - | **-0.73** | **-0.63** |
| *VPS33A* | 0.04 | **0.38** | **0.54** | **0.32** |
| *VPS39* | 0.04 | **-1.40** | **-0.20** | **-0.54** |
| *TSNARE1.1* | **2.24** | - | **1.37** | **1.02** |
| *YKT6* | -0.17 | **-0.48** | 0.10 | 0.10 |
| *USE1* | **-0.30** | **-1.90** | **0.22** | 0.10 |
| *SNAP25* | -0.01 | - | 0.17 | -0.03 |

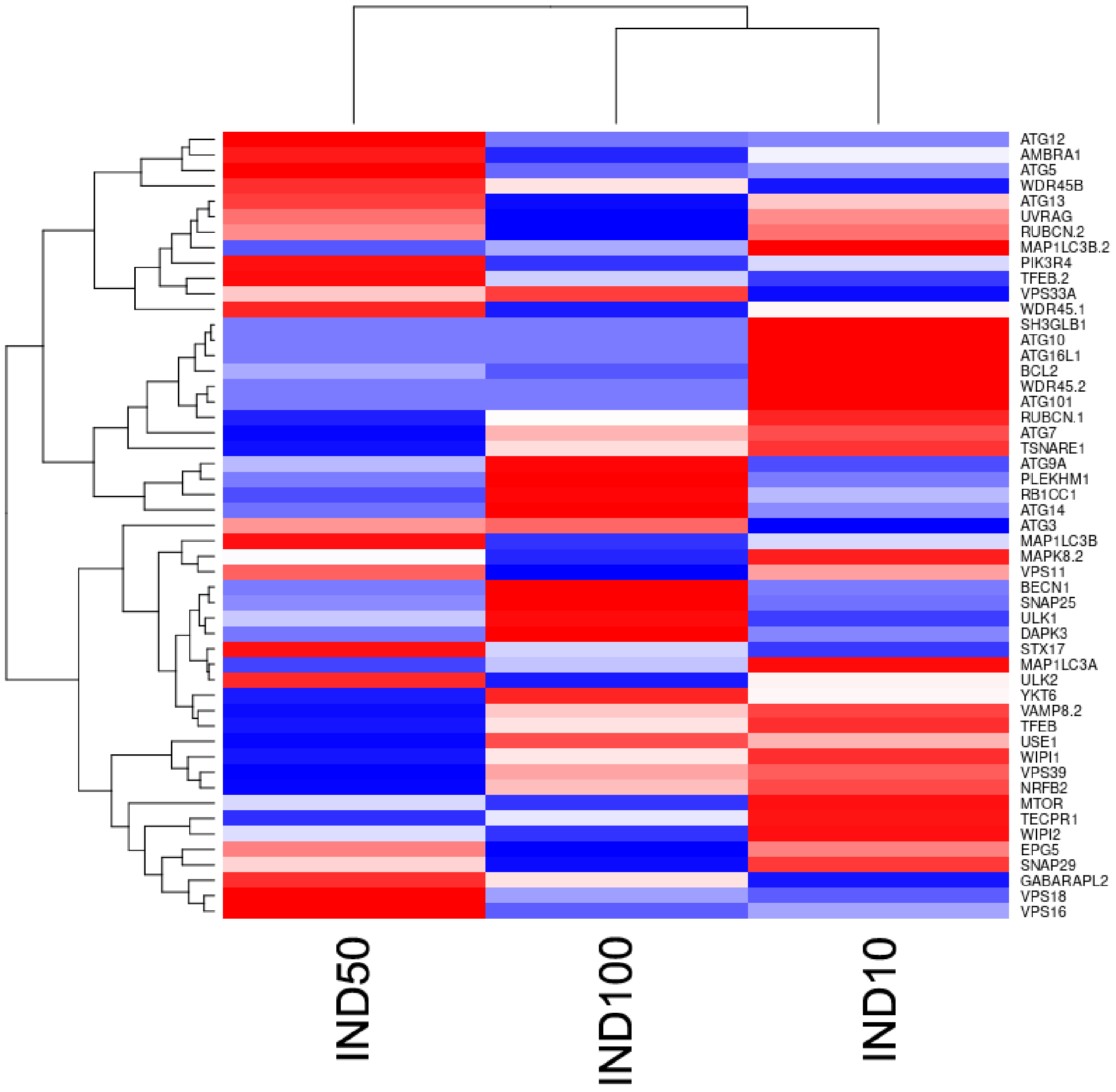


**Figure S4:** Heatmap obtained using the average values of methylation difference of the studied promoters. *PIK3C3*, *RAB7A*, *VPS41*, *INPP5E* genes were excluded due to the absence of significant data.

**Table S7.** Promoter DMRs (tiles) and their features, related to Table 1.

| Gene symbol | strand | tile chr | tile start | TSS | IND 10 | IND 50 | IND 100 | EH38 cCRE | clas-sification CGI | hg38. oreganno.id | TFBS |
|---|---|---|---|---|---|---|---|---|---|---|---|
| *MTOR* | -1 | chr1 | 11263201 | 11262551 | **30** | | | E1318739 | enhP | | |
| *ULK2* | -1 | chr17 | 19867801 | 19867936 | **24** | | **28** | **E1851797**<br>*** | prom<br>CGI | OREG1175743<br>OREG1609744<br>OREG1853806<br>OREG1482646<br>OREG0399094 | TFAP2C,<br>FOXP1,<br>RB1,<br>E2F4,<br>E2F4 |
| *BECN1* | -1 | chr17 | 42823401 | 42824282 | **26** | **49** | | | intron | | |
| *PIK3R4* | -1 | chr3 | 130747201 | 130746829 | **31** | **36** | | | up-<br>stream | OREG1230891 | SMARCA4 |
| *BCL2* | -1 | chr18 | 63320201 | 63320128 | 13 | | 3 | **E1923297**<br>** | prom<br>CGI | OREG1250101<br>OREG1219989<br>OREG1488348<br>OREG0395794<br>OREG1340917 | SMARCA4,<br>SMARCA4,<br>EGR1,<br>EGR1,<br>CTCF |
| *BCL2.2* | -1 | chr18 | 63318401 | 63318812 | **-35** | **-51** | | E1923293 | prom<br>CGI | OREG1937747<br>OREG1250101<br>OREG0396743 | VDR,<br>SMARCA4,<br>EGR1 |
| *ATG3* | -1 | chr3 | 112562801 | 112561962 | **-40** | | **-20** | E2227361<br>E2227362 | enhP | | |
| *ATG5* | -1 | chr6 | 106325201 | 106325760 | | | | E2490641<br>E2490642 | enhP/<br>prom<br>CGI | OREG1173894 | TFAP2C,<br>SMARCA4,<br>STAT1 |
| *ATG7* | 1 | chr3 | 11271401 | 11272416 | -13 | -15 | | | up-<br>stream | | |
| *WDR45.2* | -1 | chrX | 49079601 | 49080073 | | | -15 | E2754334 | enhP<br>CGI | OREG1600364<br>OREG1793948<br>OREG1823967 | FOXA1,<br>RBL2,<br>RBL2 |
| *EPG5* | -1 | chr18 | 45966201 | 45967329 | **34** | | | | intron | | |
| *VAMP8.2* | 1 | chr2 | 85562201 | 85561720 | 16 | -9 | 12 | | intron | NEAR TO<br>OREG1944064 | ZNF263 |
| *VPS11* | 1 | chr11 | 119068401 | 119067692 | **28** | **31** | | | intron | OREG1260042<br>OREG1081042 | SMARCA4,<br>TCF7L2 |
| *VPS33A* | -1 | chr12 | 122266401 | 122266494 | | | 10 | E1651497 | prom<br>CGI | OREG1250692<br>OREG1812689<br>OREG1791744<br>OREG1778450<br>OREG1220514<br>OREG1797023<br>OREG1945509<br>OREG1762962<br>OREG1612756<br>OREG1543101<br>OREG1139438<br>OREG1112385<br>OREG0231119<br>OREG1112386 | SMARCA4,<br>RBL2,<br>RBL2,<br>RBL2,<br>SMARCA4,<br>RBL2,<br>ZNF263,<br>MITF,<br>FOS,<br>ETS1,<br>ZNF263,<br>USF2,<br>USF1,<br>USF2 |

| | | | | | | | | | | | |
|---|---|---|---|---|---|---|---|---|---|---|---|
| *MAPK8.2* | 1 | chr10 | 48306201 | 48306743 | | | | **E1466099**<br>** | prom<br>CGI | OREG1493767<br>OREG1173286<br>OREG1766204<br>OREG0273979<br>OREG0273980<br>OREG0367292 | EGR1,<br>TFAP2C,<br>MITF,<br>ZEB1,<br>ZEB1,<br>EBF1 |
| *MAP1LC3A* | 1 | chr20 | 34558801 | 34558718 | 14 | 12 | 15 | E2108528 | enhP<br>CGI | | |
| *SNAP25 (a)* | 1 | chr20 | 10217801 | 10218830 | | | 12 | E2097209 | enhP<br>CGI | | |
| *SNAP25 (b)* | 1 | chr20 | 10219601 | 10218830 | | -13 | 11 | E2097215 | prom | OREG1893190<br>OREG1181996<br>OREG1844288<br>OREG1848497<br>OREG1856839<br>OREG1232693<br>OREG1485028<br>OREG0004318<br>OREG0004317<br>OREG1100322 | STAT1,<br>TFAP2C,<br>RB1,<br>RB1,<br>RB1,<br>SMARCA4,<br>E2F4,<br>REST,<br>REST,<br>TFAP2C |

UCSC hg38 functional annotations:

EH38 CCRE: ENCODE Candidate Cis-Regulatory Elements (cCREs) combined from all cell types [1]:

enhP: proximal enhancer-like signature

prom: promoter-like signature

** DNase Z-score, H3K4me3 Z-score >1.64 in CaCo-2 cells

*** DNase Z-score, H3K4me3 Z-score, CTCF Z-score >1.64 in CaCo-2 cells.

ORegAnno: Regulatory elements from ORegAnno [2].

Abbreviations:

TSS: Transcription Start Site

TFBS: Transcription Factor Binding Site

CGI: CpG Island

**Table S8.** Gene body DMRs (tiles) and their features, related to Table 2.

| **Gene symbol** | **strand** | **tile chr** | **tile start** | **IND 10** | **IND 50** | **IND 100** | **EH38 cCRE** | clas-sification CGI | hg38. oreganno.id | TFBS |
|---|---|---|---|---|---|---|---|---|---|---|
| MTOR | -1 | chr1 | 11119401 | 13 | | -22 | | intron | | |
| | | chr1 | 11122601 | | **-47** | | | intron | | |
| | | chr1 | 11193601 | -13 | -10 | -17 | E1318692 | enhD | ANGPTL7 exon | |
| | | chr1 | 11250001 | | | 23 | | intron | | |
| | | chr1 | 11261201 | | -23 | | | intron | | |
| ULK1 | 1 | chr12 | 131896401 | 14 | | | **E1657097 **** | prom | OREG1193847 OREG1601983 OREG1660293 OREG1824583 OREG1789197 OREG1854905 OREG0833612 OREG0986170 OREG0880598 OREG1809256 OREG1846437 OREG1843192 | ULK1, TFAP2C, FOXA1, RBL2, RB1, MAFF, NFE2::MAF, MAFK, RB1 |
| | | chr12 | 131908201 | | | 17 | | intron CGI | | |
| | | chr12 | 131909401 | | -17 | | | intron CGI | | |
| | | chr12 | 131919801 | | | **-26** | | intron | | |
| ULK2 | -1 | chr17 | 19772601 | | | | | intron | | |
| | | chr17 | 19772801 | | -20 | -21 | | intron | near to E1851762 | |
| | | chr17 | 19836201 | 16 | 20 | 14 | | intron | | |
| | | chr17 | 19838801 | | | | | intron | | |
| | | chr17 | 19842401 | | | 24 | | intron | | |
| ATG9A | -1 | chr2 | 219219401 | 15 | | **51** | E2075977 | enhP | near to ABCB6 promoter | |
| PIK3R4 | -1 | chr3 | 130727401 | | **74** | | E2238076 E2238077 | enhD | | |
| AMBRA1 | -1 | chr11 | 46429001 | | | 13 | | intron | H3K27Ac | |
| | | chr11 | 46520601 | | 21 | | | intron | | |
| | | chr11 | 46548201 | | 17 | 19 | | exon2 | | |
| | | chr11 | 46553001 | | | | | intron | | |
| | | chr11 | 46566201 | | -16 | | | intron | | |
| UVRAG | 1 | chr11 | 75877401 | 20 | 20 | 22 | | intron | | |
| | | chr11 | 75887401 | -8 | | -18 | | intron | | |
| | | chr11 | 75974401 | 24 | | | | intron | | |
| ATG7 | 1 | chr3 | 11290801 | -22 | | | E2178358 | enhP | OREG1616328 OREG0665690 OREG0741992 | ATG7,FOS, FOSL1, JUNB |

| | | | | | | | | | | |
|---|---|---|---|---|---|---|---|---|---|---|
| | | chr3 | 11379801 | **-37** | | **-29** | E2178411 | enhD | OREG1256740 OREG0563863 OREG0730235 OREG1675345 | ATG7,SMARCA4, DUX4, JUN, ATG7,GATA2 |
| ATG10 | 1 | chr5 | 82037401 | -18 | | | | intron | | |
| MAP1LC3B | 1 | chr16 | 87390401 | -17 | -15 | | | intron | | |
| WIPI2 | 1 | chr7 | 5222801 | -11 | | | **E2531309** ** | enhD/intron | OREG1602525 OREG1660808 OREG1194239 | WIPI2,FOXA1, TFAP2C |
| WDR45B | -1 | chr17 | 82646801 | 17 | | | E1893683 | intron | | |
| | | chr17 | 82647001 | | | 21 | E1893683 | intron | | |
| WDR45.1/.2 | -1 | chrX | 49099601 | **30** | | 24 | | intron | H3K27Ac in K562 | |
| RUBCN.1/.2 | -1 | chr3 | 197707201 | | | -12 | | intron | | |
| | | chr3 | 197729201 | | -9 | -24 | | intron | | |
| RUBCN.2 | -1 | chr3 | 197739401 | | -20 | | E2273633 E2273634 | intron | | |
| RAB7A | 1 | chr3 | 128743201 | 14 | | | | intron | OREG1926731 | TRIM28 |
| PLEKHM1 | -1 | chr17 | 45437001 | 10 | | | | last exon | OREG0844941 OREG0890141 | MAFF, MAFK |
| | | chr17 | 45453401 | | | -20 | E1866153 | exon | | |
| | | chr17 | 45485801 | | -17 | | | intron | | |
| EPG5 | -1 | chr18 | 45966201 | **34** | | | | intron | | |
| INPP5E | -1 | chr9 | 136430201 | | | -12 | E2736673 | enhD | | |
| | | chr9 | 136432601 | | | | | intron CGI | | |
| | | chr9 | 136432801 | -18 | -20 | | | intron | OREG1510897 | EGR1 |
| SNAP29 | 1 | chr22 | 20860401 | -24 | -14 | -22 | | intron | OREG1489467 OREG1946017 | PI4KA,EGR1, TOP3B, ZNF263 |
| VAMP8.2 | 1 | chr2 | 85562801 | | 21 | 20 | | intron | | |
| | | chr2 | 85563001 | | | 11 | | intron | | |
| | | chr2 | 85563201 | -12 | | -16 | | intron | | |
| | | chr2 | 85563601 | | -10 | | | intron | OREG1303251 | CEBPA |
| VPS11 | 1 | chr11 | 119072401 | 14 | | | | intron | | |
| VPS18 | 1 | chr15 | 40899601 | | 16 | 15 | | exon | | |
| | | chr15 | 40899801 | -19 | | | | exon | | |
| VPS33A | -1 | chr12 | 122241201 | | 11 | | | intron | | |
| MAPK8.2 | 1 | chr10 | 48346401 | | | | E1466129 | CTCF | | |
| DAPK3 | -1 | chr19 | 3964801 | | | | | exon CGI | OREG1505997 | EGR1 |
| GABARAPL2 | 1 | chr16 | 75571801 | | -18 | **-38** | E1828346 | intron | | |
| TSNARE1.1/.2 | -1 | chr8 | 142237601 | | 18 | | | intron | OREG1516995 | EGR1 |
| | | chr8 | 142243201 | -20 | | | | intron | | |
| | | chr8 | 142244001 | | | | | intron | | |

| | | | | | | | | | | |
|---|---|---|---|---|---|---|---|---|---|---|
| | | chr8 | 142249201 | | **-27** | | | intron | | |
| | | chr8 | 142252001 | | | -17 | | intron CGI | | |
| | | chr8 | 142253001 | | 21 | | | intron | | |
| | | chr8 | 142253201 | | 17 | | | intron | | |
| | | chr8 | 142257201 | | | | | intron | | |
| | | chr8 | 142261601 | **37** | | | | intron | OREG1946662 | ZNF263 |
| | | chr8 | 142271201 | **61** | **63** | **28** | | exon or intron | OREG1517008 | EGR1 |
| | | chr8 | 142325801 | | -16 | | E2674338 E2674339 | enhD CGI | | |
| | | chr8 | 142326001 | | | 11 | E2674339 | enhD CGI | | |
| | | chr8 | 142326201 | | 13 | 13 | E2674339 | enhD CGI | | |
| | | chr8 | 142326401 | -15 | | | E2674339 | enhD CGI | | |
| | | chr8 | 142346401 | | -16 | -19 | E2674353 | K4m3 | | |
| TSNARE.1 | -1 | chr8 | 142361001 | -11 | | | | intron | | |
| | | chr8 | 142375201 | | | **36** | | intron | | |
| SNAP25 | 1 | chr20 | 10278801 | | | 15 | | intron | | |

UCSC hg38 functional annotations:

EH38 CCRE: ENCODE Candidate Cis-Regulatory Elements (cCREs) combined from all cell types [1]:

enhP: proximal enhancer-like signature

enhD: distal enhancer-like signature

K4m3: DNase-H3K4me3

CTCF: CTCF-only

** DNase Z-score, H3K4me3 Z-score >1.64 in CaCo-2 cells.

ORegAnno: Regulatory elements from ORegAnno [2].

Abbreviations:

TFBS: Transcription Factor Binding Site

CGI: CpG Island.

**References:**


1. ENCODE Project Consortium. Expanded Encyclopedias of DNA Elements in the Human and Mouse Genomes. Nature. 2020 July 30;583(7818):699-710. DOI: 10.1038/s41586-020-2493-4
2. Lesurf R, Cotto KC, Wang G, Griffith M, Kasaian K, Jones SJ, Montgomery SB, Griffith OL, Open Regulatory Annotation Consortium. ORegAnno 3.0: a community-driven resource for curated regulatory annotation. Nucleic Acids Res. 2016 Jan 4;44(D1):D126-32. DOI: 10.1093/nar/gkv1203
